# Choice of Acromioclavicular Dislocation Treatment Should Not Be Influenced by Risk of Development of Acromioclavicular Osteoarthritis: A Systematic Review With Meta-Analysis

**DOI:** 10.2106/JBJS.RVW.24.00085

**Published:** 2024-09-03

**Authors:** Roderick J.M. Vossen, Daniel Verstift, Faridi S. Van Etten-Jamaludin, Bryan J. Hamans, Michel P.J. van den Bekerom, Lukas P.E. Verweij

**Affiliations:** 1 Department of Orthopedics, OLVG, Amsterdam, the Netherlands; 2 Amsterdam Shoulder and Elbow Centre of Expertise (ASECE), Amsterdam, the Netherlands; 3 Department of Orthopedic Surgery and Sports Medicine, Amsterdam UMC, location AMC, University of Amsterdam, Amsterdam, the Netherlands; 4 Medical Library AMC, Amsterdam UMC location University of Amsterdam, Amsterdam, the Netherlands; 5 Department of Human Movement Sciences, Faculty of Behavioral and Movement Sciences, Vrije Universiteit Amsterdam, Amsterdam Movement Sciences, Amsterdam, the Netherlands; 6 Musculoskeletal Health Program, Amsterdam Movement Sciences, Amsterdam, the Netherlands

## Abstract

**Background:**

It is currently unknown to what degree surgical or nonoperative treatment of acromioclavicular (AC) dislocation influences the development of osteoarthritis (OA). The aim of this study was to evaluate AC OA after surgical and nonoperative treatment for AC dislocations, compare OA prevalence between treatment options, and compare OA prevalence between the injured and contralateral shoulder.

**Methods:**

Articles reporting on the prevalence of OA after surgical or nonoperative treatment of an AC dislocation with a minimal 2-year follow-up were included. AC OA presence was extracted for the injured and contralateral shoulder. Treatment categories were defined based on anatomical variation in the reattachment of ligaments: AC fixation, coracoclavicular (CC) fixation, AC and CC fixation, Bosworth screw synthetic graft, tendon graft, and conservative. Study quality was assessed using the Methodological Index for Non-Randomized Studies (MINORS) criteria.

**Results:**

Ninety-four articles were included for qualitative analysis, and 7 articles were included for meta-analysis (n = 3,812; follow-up = 2.0-24.2 years; mean age 37.6 ± 10.4 years). A total of 3,483 patients underwent surgical treatment, and 329 patients underwent conservative treatment. OA prevalence ranged from 6.7%-29.3% between 7 pooled treatment categories. Most included studies had a follow-up <10 years (94%) and OA prevalence increased with time, regardless of treatment option. There was no difference in OA prevalence between the injured and contralateral shoulder (p = 0.120). MINORS scores were varied, ranging from poor to very good.

**Conclusion:**

The pooled AC OA prevalence of the 7 treatment categories ranged from 6.7% for the CC fixation surgical group to 29.3% for the conservative treatment group. However, the included studies were predominantly of low quality and had varying follow-up periods, with most having relatively short follow-up durations. No difference in AC OA prevalence was found between the injured and contralateral shoulder. Based on the available evidence, treatment choice for AC dislocation should not be influenced by the potential development of AC AO.

**Level of Evidence:**

Level IV. See Instructions for Authors for a complete description of levels of evidence.

An acromioclavicular (AC) dislocation is primarily caused by a direct force to the AC joint in combination with the arm in adduction^1^. Patients suffering from AC dislocation typically experience pain located in the superior-anterior region of the shoulder that increases during cross-body activities^2^.

AC dislocations can be classified using the Rockwood classification, which consists of 6 types that are based on the injured ligaments and direction of displacement of the clavicle, which helps to grade severity. Tamaoki et al. demonstrated that surgical treatment for AC dislocation in adults had no additional benefits in terms of function, return to former activities, and quality of life at 1 year postoperatively^3^. Nevertheless, long-term studies comparing surgical treatments generally find contradicting conclusions, leading to a lack of consensus for the optimal treatment.

Osteoarthritis (OA) of the AC joint consists of joint space narrowing, osteophyte formation, subchondral sclerosis and cysts and can be symptomatic in a subset of patients^3-6^. Some literature suggests that AC OA is a common, late-onset complication of AC dislocation^6^. Greiner et al. showed that patients may have a higher risk of AC OA after an AC dislocation compared with the contralateral shoulder^7^. However, because of the high prevalence of asymptomatic AC OA in the general healthy population and the lack of clear reporting on complications, the risk of AC OA development after treatment of AC dislocation remains unclear^8^.

Two radiographic studies By Bonsell et al. and Pennington et al. both reported an approximate prevalence of asymptomatic AC OA of 45.0% in a healthy population between age 40 and 83 years^9,10^. A recent systematic review by Rossano et al. evaluated the prevalence of asymptomatic AC OA in a healthy population and included predominantly magnetic resonance imaging (MRI)–based studies. This study demonstrated a prevalence of asymptomatic AC OA of up to 70%, increasing with age^8^.

It is currently unknown to what degree surgical or nonoperative treatment of AC dislocation influences the development of AC OA. The aim of this systematic review was to evaluate the prevalence of AC OA after surgical and nonoperative treatment for AC dislocations, to compare AC OA prevalence between treatment options, and to compare AC OA prevalence between the injured and contralateral uninjured shoulder. It was hypothesized that postoperative AC OA prevalence would not significantly differ between the surgical treatment groups but that the conservative treatment group would demonstrate the highest prevalence of AC OA.

## Methods

### Study Design

This systematic review with meta-analysis was conducted according to the Preferred Reporting Items for Systematic Reviews and Meta-analyses guidelines^11^. All RCTs, cohort studies, case-control studies and case series reporting on OA after surgical or nonoperative treatment of AC dislocation until June 2023 were eligible for inclusion. Studies were included in case they (1) reported on the OA prevalence for any Rockwood classification and (2) had a minimum follow-up of 2 years. Studies were excluded in case they (1) included patients younger than 18 years old, (2) reported nonoriginal data, (3) had a sample size <5 patients, (4) solely reported on postoperative AC OA after a distal clavicle resection, (5) were cadaver or animal studies, and (6) used a language other than English, German, French, or Dutch.

### Literature Search

A literature search was conducted to identify studies using electronic databases: PubMed/Medline (B.V. Elsevier), Embase (Ovid) (B.V. Elsevier), and Cochrane Central (B.V. Wiley). The search strategy, including the search terms, was listed (Appendix 1). The abstracts of the retrieved articles were independently screened for eligibility by 3 independent researchers (R.J.M.V., D.V., and B.J.H.) using Rayyan (B.V. Qatar Computing Research Institute)^12^. The full-text articles of potential studies were assessed for eligibility by 3 reviewers (R.J.M.V., D.V., and B.J.H.). Any disagreement was resolved by discussion and consensus.

### Data Collection

Study characteristics, including sample size, mean age, Rockwood type, lateralization, and treatment type, were extracted. OA prevalence of the injured and contralateral (uninjured) shoulder was extracted. As the OA definition was expected to vary or not be reported, the OA prevalence was directly adopted from the included studies according to their definition. In addition, each included article was screened for a used definition for AC OA, which was extracted when reported. Two independent reviewers (R.J.M.V. and B.J.H.) extracted the data from the included studies. To ensure the accuracy and completeness of the data, a third reviewer (D.V.), verified the extracted data.

### Treatment Categorization

As a large variety of treatment options were expected, a comparison for surgical vs. nonoperative treatment was not feasible. To get insight into possible differences, operative treatments were pooled based on the anatomical variation in the reattachment of ligaments and material used because these are often similar. If multiple treatment options were addressed in 1 article, each treatment option and the corresponding data were placed accordingly in each category. Treatment options were categorized in 7 categories, which included AC fixation, defined as a surgical intervention stabilizing the AC ligament; coracoclavicular (CC) fixation, defined as a surgical intervention stabilizing the CC ligaments; AC and CC fixation, defined as surgical options stabilizing both the AC and CC ligaments; and the Bosworth Screw, defined as treatment option using a screw to fixate the clavicle to the acromion. All treatment options using a tendon or synthetic graft were categorized in the tendon or synthetic graft groups, respectively. Treatment options that did not use any form of surgical intervention were included in the nonoperative group.

### Quality Assessment

A quality appraisal was performed using the Methodological Index for Non-Randomized Studies (MINORS) criteria^13^. MINORS is a validated tool for assessing the methodological quality of nonrandomized studies looking at the clarity of the research question and data collection methods. MINORS scores were used to assess the risk of bias and to determine the overall quality of the evidence.

### Statistical Analysis

Patient characteristics and follow-up were pooled by calculation of weighted means and pooled SDs according to Walter et al.^14^. If the mean was not reported, it was estimated according to Hozo et al.^15^. OA prevalence was presented for individual studies. Owing to the heterogeneity of data, statistical comparisons were not feasible between treatment categories. Therefore, scatterplots were created with the OA prevalence (y-axis) per individual study and follow up-duration (x-axis) to explore potential patterns. Pooled OA prevalence was compared by combining the data of treatment categories. A meta-analysis was performed between treatment options if at least 2 comparative studies compared AC OA between the injured and contralateral shoulder. Review Manager v5.3 (the Nordic Cochrane Center, Copenhagen, Denmark) was used to calculate risk ratios with 95% confidence interval. Total OA prevalence and OA prevalence of each treatment category in the meta-analysis was compared by use of an χ^2^ test and a random-effects model. Heterogeneity between studies was assessed using the I^2^ statistic. I^2^ > 50% was considered as substantial heterogeneity^16^. Statistical level of significance was set to p < 0.05. Number of articles that defined AC OA was expressed in percentages.

## Results

### Study Selection and Characteristics

**Fig. 1:**
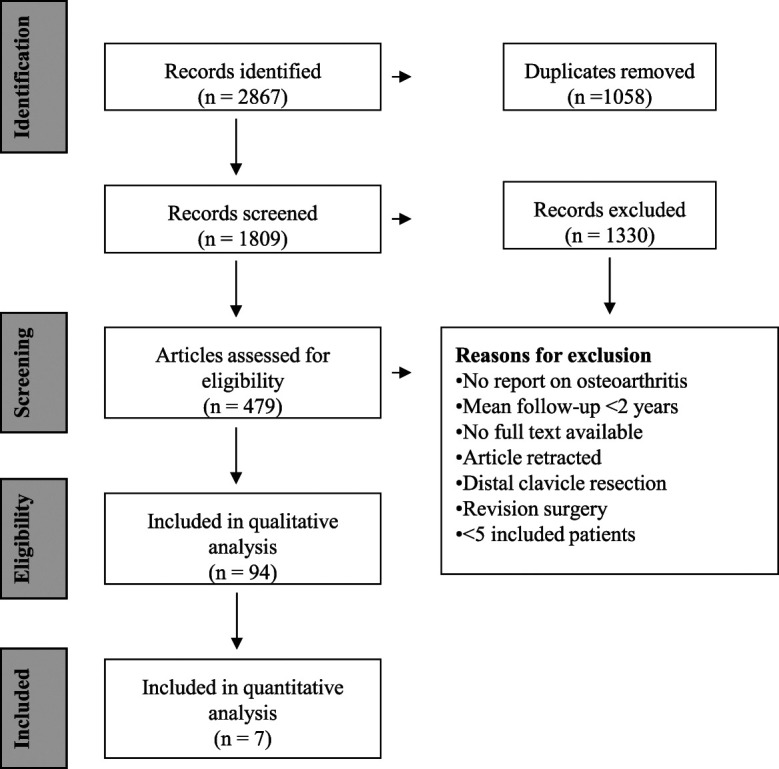
PRISMA flowchart of the study selection.

A total of 94 articles were included for qualitative analysis and 7 articles were included for quantitative meta-analysis. Reasons for exclusion were listed (Fig. 1). Included articles comprised 3,812 patients with a mean age of 37.6 ± 10.4 years and a follow-up ranging from 2.0 years to 24.2 years. Six articles reported a mean follow-up of >10 years (6%). The overall OA prevalence ranged from 0% to 100% and was reported for 59 different treatment types: 54 surgical treatment types and 5 conservative treatment types. MINORS scores were varied, ranging from poor to very good (Table I).

**TABLE I:** Study Characteristics*†

First Author (Year)	Design	MINORS Score	Sample Size	Male (%)	Age at Luxation, yr Mean ± SD	Mean FU, mo	RW Type	Procedure	Procedure Category	OA (%)
Ko SH (2023)^17^	R	16/24	61	88.5	51.5 ± 13.1	84.0	III, IV, V	Hookplate Tightrope	AC fixation CC fixation	11.1 4.0
Eckl L (2023)^18^	R	22/24	28	—	37.8	30.5	III, IV, V	Single suture button Double suture button	AC and CC fixation AC and CC fixation	21.4 39.3
Huang J (2023)^19^	R	20/24	72	77.8	27.8	32.0	III, V	Tightrope	CC fixation	0
Liu G (2022)^20^	R	19/24	57	68.4	38.8 ± 11.8	46.2	V	Hookplate	AC fixation	1.8
Madi S (2022)^21^	R	23/24	32	90.6	44.3 ± 12.2	32.9	III, V	Hookplate Fibertape	AC fixation CC fixation	19.0 6.0
Verstift D (2021)^22^	R	20/24	72	83.3	43.0	85.8	I, II	Sling/taping	Conservative	44.0
Gu F (2021)^23^	R	21/24	66	63.6	44.7 ± 14.9	49.0	IV, V	Single and double tightrope	AC fixation	2.0
Pattu R (2021)^24^	P	8/24	17	76.5	37.0 ± 7.6	30.0	V	Endobutton + suture	AC- and CC fixation	0
Yoo YS (2021)^25^	R	22/24	22	68.5	43.6 ± 5.9	32.0	III, V	Hookplate Anchor knots	AC fixation CC fixation	40.0 0
Motta P (2020)^26^	R	9/16	14	85.7	36.0	91.0	IV	Graftrope (allograft)	Tendon	21.0
Cerciello S (2020)^27^	R	10/16	42	80.9	42.7 ± 12.8	45.6	III, V	ACCR (allograft)	Tendon	7.0
Cano-Martínez JA (2020)^28^	R	10/16	21	90.5	30.7 ± 11.7	49.7	III, V	Triple tightrope	CC fixation	14.0
Ozan F (2020)^29^	R	12/16	24	75.0	41.8 ± 11.7	42.0	III	Tension band	AC fixation	21.0
Breuer R (2019)^30^	R	8/16	65	95.4	42.0 ± 9.3	57.3	III-V	MINAR	CC fixation	8.0
Muench LN (2019)^31^	R	10/16	43	76.7	43.4 ± 11.4	40.8	III, V	ACCR (allograft)	Tendon graft	7.0
Bigoni M (2019)^32^	R	8/16	16	93.8	46.0	28.8	III, V	Tapes + K-wire	AC fixation	6.0
Sun LJ (2019)^33^	R	16/26	80	67.5	45.4 ± 12.3	34.4	II-V	Single tightrope Double tightrope	CC fixation CC fixation	0 0
Tiefenboeck TM (2018)^34^	R	10/16	47	93.6	37.0 ± 10.7	89.0	III-V	LARS	Synthetic	13.0
Issa SP (2018)^35^	R	9/16	19	84.2	34.4 ± 8.3	76.9	III, IV	Tightrope	CC fixation	26.0
Xue C (2018)^36^	P	12/16	25	60.0	43.0 ± 14.6	34.0	V	Endobutton + CC reconstruction	CC fixation	14.0
Hashiguchi H (2018)^37^	R	10/16	12	100	40.8 ± 13.2	106.3	III, V	ACCR (synthetic)	Synthetic	17.0
Moura DL (2018)^38^	R	10/16	153	91.5	29.2 ± 9.5	55.4	III-V	HUC	AC fixation	0
Mori D (2017)^39^	P	10/16	20	90.0	32.3 ± 16.3	151.8	III-V	ACCR (synthetic)	Synthetic	90.0
Tiefenboeck TM (2017)^40^	R	8/16	22	90.9	41.0 ± 15.6	93.6	II-V	Bosworth screw	Bosworth	18.0
Garofalo R (2017)^41^	R	11/16	32	75.0	32.4 ± 7.1	29.2	V	Autograft + CC reconstruction	Tendon	0
Cetinkaya E (2017)^42^	R	14/24	32	78.2	45.7 ± 11.4	94.5	III	Bosworth screw K-wire	Bosworth AC fixation	12.0 18.0
Horst K (2017)^43^	R	16/24	42	93.6	37.5 ± 11.9	58.15	III	K-wire K-wire + ligament transfer Single tightrope Double tightrope	AC fixation AC fixation CC fixation CC fixation	17.0 0 0 8
Dal Molin F (2017)^44^	P	10/16	20	95.0	34.8 ± 11.7	45.0	IV, V	CC-cerclage	CC fixation	20.0
Takase K (2016)^45^	P	12/16	22	86.4	38.1 ± 13.4	38.0	V	ACCR (tendon)	Tendon	5.0
Lateur G (2016)^46^	R	12/24	25	92.0	35.1 ± 11.0	150.0	IV, V	K-wire	AC fixation	47.0
Metzlaff S (2016)^47^	P	10/24	44	—	37.0 ± 8.8	34.8	III-V	Hookplate MINAR	AC fixation CC fixation	0 0
Cano-Martinez JA (2016)^48^	R	6/16	33	78.8	25.0 ± 7.0	25.0	V	MINAR	CC fixation	6.0
Lu D (2016)^49^	P	11/24	80	68.8	33.9 ± 11.8	25.4	III-V	Double endobutton Triple endobutton	CC fixation CC fixation	0 0
McKee (2015)^50^	P	24/24	83	93.8	37.6	24.0	III-V	Hookplate Sling + physiotherapy	AC fixation Conservative	10.0 2.0
Shin S (2015)^51^	P	14/16	18	94.4	45.4 ± 11.9	25.6	III-V	Single tightrope	CC fixation	6.0
Loriaut P (2015)^52^	R	13/16	39	66.7	35.7 ± 8.1	42.3	III, IV	Double tightrope	CC fixation	0
Vascellari A (2015)^53^	R	7/24	14	91.7	45.0	50.9	III	Tightrope Graftrope (allograft) LARS	CC fixation Tendon Synthetic	25.0 20.0 75.0
Natera Cisneros L (2015)^54^	R	21/24	41	85.4	37.0	36.0	III-V	Sling Single tightrope	Conservative CC fixation	0 5.0
Rosslenbroich SB (2015)^55^	R	14/16	83	96.4	39.0	39.0	III-V	MINAR	CC fixation	0
Liu X (2015)^56^	R	5/16	12	66.7	48.0 ± 10.4	24.0	III, V	CC ligament augmentation	CC fixation	0
Ye T (2014)^57^	R	6/16	39	87.2	36.0 ± 6.9	42.0	III-V	Cables	CC fixation	23.0
Cerciello S (2014)^58^	P	7/16	28	92.9	31.0 ± 7.8	72.0	III	Cadenat	AC fixation	0
Joukainen A (2014)^59^	P	21/24	25	91.4	53.5 ± 8.3	226.8	III-V	K-wire + suture Splint	AC fixation Conservative	69.0 100
Wang Y (2014)^60^	R	5/16	21	95.2	41.6 ± 9.9	33.0	III, V	Hookplate	AC fixation	14.0
Virtanen KJ (2014)^61^	R	13/16	25	84.0	44.0	50.4	II-V	Autograft + suture	Tendon	4.0
Lu N (2014)^62^	R	5/16	24	66.7	31.0	36.0	III-V	LARS	Synthetic	8.0
Kraus N (2013)^63^	P	20/24	28	92.8	39.3 ± 12.6	24.0	V	Single tightrope Double tightrope	CC fixation CC fixation	27.0 23.0
Wang Y (2013)^64^	P	5/16	21	90.5	45.0 ± 11.0	32.1	V	Dewar	AC fixation	5.0
Virtanen KJ (2013)^65^	R	13/16	50	84.0	36.0 ± 8.2	215.0	V	K-wire	AC fixation	33.0
Sandmann GH (2012)^66^	R	8/16	33	90.9	39.0 ± 13.0	32.0	III-V	PDS	AC and CC fixation	18.0
Motta P (2012)^67^	R	11/16	51	98.0	36.0 ± 10.2	64.8	III-V	LARS	Synthetic	37.0
Kim SH (2012)^68^	R	10/16	12	100	37.3 ± 7.7	31.2	V	CAL transfer	Tendon	67.0
Verdano MA (2012)^69^	R	12/16	14	78.6	44.0	35.1	III-V	K-wire	AC fixation	14.0
Liu Q (2012)^70^	R	11/16	16	62.5	36.0	26	III	MAAP	AC fixation	5.0
Eschler A (2012)^71^	R	10/16	52	—	42.3	32.0	V	Hookplate PDS	AC fixation AC and CC fixation	110. 8.0
El Shewy MT (2011)^72^	P	7/16	21	76.2	31.8 ± 4.6	92.4	IV, V	Suture	CC fixation	0
Takase K (2011)^73^	R	16/24	138	91.6	35.5 ± 10.4	40.0	V	Cadenat Dewar	AC fixation AC fixation	12.0 36.0
Ladermann A (2011)^74^	R	12/16	37	94.6	33.6 ± 8.9	54.0	III-V	PDS	AC and CC fixation	19.0
Lizaur A (2011)^75^	P	13/16	38	94.7	32.4 ± 10.6	290.4	III	K-wire	AC fixation	29.0
Assaghir YM (2011)^76^	R	10/16	56	85.6	35.2 ± 7.3	74.6	III-V	CC lag screw	Bosworth	2.0
Kienast B (2011)^77^	R	12/16	225	—	38.4	36.0	III-V	Hookplate	AC fixation	14.0
Salzmann GM (2010)^78^	P	12/16	23	91.3	37.5 ± 10.2	30.6	III-V	Double tightrope + K-wire	AC and CC fixation	0
Mares O (2010)^79^	R	13/16	27	88.9	43.0	60.0	III-V	Synthetic ligament reconstruction	Synthetic	7.0
Esenyel CZ (2010)^80^	R	11/16	32	75.0	35.0	37.2	III-VI	Bosworth screw	Bosworth	0
Murena L (2009)^81^	P	13/16	16	93.8	33.3 ± 9.6	31.0	III-V	Endobutton + K-wire	CC fixation	0
Greiner S (2009)^7^	R	12/16	50	86.0	35.3 ± 10.2	70.0	III-V	PDS	AC and CC fixation	36.0
Shin S (2009)^51^	R	12/16	29	89.7	39.7 ± 9.3	27.8	V	Suture + AC ligament transfer	CC fixation	7.0
Salem KH (2009)^82^	R	14/16	25	92.0	41.0	30.0	III-V	Hookplate	AC fixation	8.0
Choi SW (2008)^83^	R	11/16	20	80.0	33.5 ± 12.1	41.2	III-V	Suture	CC fixation	5.0
Gstettner C (2008)^84^	R	21/24	41	—	36.7 ± 11.6	34.5	III	Hookplate Sling	AC fixation Conservative	51.0 100
Dimakopoulos P (2006)^85^	R	10/16	34	91.2	33.5	33.2	III, V	CC stabilisation	CC fixation	0
Fremerey R (2005)^86^	R	14/24	80	—	34.8 ± 11.8	75.6	III, V	PDS Physiotherapy	AC and CC fixation Conservative	12.0 11.0
Mouhsine E (2003)^87^	P	11/16	33	84.8	25.0 ± 2.2	75.6	I, II	Sling	Conservative	54.0
Tienen TG (2003)^88^	P	12/16	21	—	32.6 ± 8.6	35.7	V	PDS	AC and CC fixation	0
Pavlik A (2001)^89^	R	10/16	17	92.3	37.2	36.6	III	Bosworth screw + Weaver-Dunn	Bosworth	18.0
Hessmann MH (2001)^90^	R	9/16	52	76.9	36.8	55.2	III	Hookplate	AC fixation	41.0
Mönig SP (1999)^91^	R	4/16	48	79.2	33.0	39.5	III	PDS	AC and CC fixation	17.0
Hessmann MH (1997)^92^	R	9/16	55	—	40.8	28.0	III-V	PDS	AC and CC fixation	5.0
Broos P (1997)^93^	R	15/24	87	—	40.0	51.6	III	Hookplate Bosworth screw	AC fixation Bosworth	43.0 39.0
Sim E (1995)^94^	R	12/16	21	100	31.0	38.0	III, V	Hookplate	AC fixation	29.0
Hessmann MH (1995)^95^	R	16/16	64	—	42.0	32.0	III-V	PDS	AC and CC fixation	6.0
Haaker R (1994)^96^	R	16/24	22	100	21.4	28.8	III-VI	Threads Threads	AC and CC fixation CC fixation	19.0 33.0
Mulier T (1993)^97^	P	12/16	58	—	31.0 ± 7.1	76.8	III-V	Sling/taping	Conservative	21.0
Verhaven E (1993)^98^	P	11/16	18	—	27.0 ± 4.4	72.0	V	CC cerclage	CC fixation	33.0
Sundaram N (1993)^99^	P	12/16	31	90.3	32.5 ± 10.3	139.2	III	Bosworth screw	Bosworth	19.0
Verhaven E (1992)^100^	P	11/16	28	—	37.5	61.2	V	CC cerclage	CC-fixation	21.0
Jalovaara P (1991)^101^	R	8/16	55	90.9	34.0 ± 10.4	78.0	III	Knowles pin	AC fixation	26.0
Eskola A (1991)^102^	R	16/24	70	—	37.0	48.0	I-III	K-wire K-wire ASIF screw	AC fixation AC fixation Bosworth	7.0 20.0 190.
Fröhling M (1989)^103^	R	8/16	21	80.9	33.0	27.0	III	Bosworth screw	Bosworth	44.0
Karlsson J (1986)^104^	R	6/16	47	89.4	26.8	72.0	III	CC ligament transfer	CC fixation	4.0
Paavolainen P (1983)^105^	R	9/16	39	94.9	42.5	48.0	III	ASIF screw	Bosworth	10.0
Lowe GP (1977)^106^	R	10/16	21	95.2	23.0 ± 9.4	120.0	III	Bosworth screw	Bosworth	10.0
Ejeskär A (1974)^107^	R	11/16	65	92.3	39.0 ± 10.3	115.2	I-VI	Cerclage	CC fixation	11.0
Weitzman G (1967)^108^	R	7/16	19	100	27.0 ± 8.7	34.0	I-VI	Bosworth screw	Bosworth	11.0

* Study characteristics were presented for each included study, recognized by the first author and publication year. Study design is defined as R; retrospective or P; prospective. MINORS criteria were presented as scored points of total, depending on the type of study. The treatment procedure or procedures were presented, together with the treatment category the study was included in. Reported percentages of patients with OA were presented.

† FU = follow-up; OA = osteoarthritis, and RW = Rockwood grade.

### Treatment Categories and Pooled AC OA Prevalence

Patients were categorized in predefined treatment categories. After categorization, 1,199 patients from 29 studies were included in the AC fixation category^19-21,23,25,29,38,42,43,46,47,58,59,64,65,69-71,73,75,77,82,84,90,93,94,101,109^, 1,050 patients from 32 studies were included in the CC fixation category^17,19,25,28,30,33,35,36,43,44,47-49,51-57,63,72,81,83,85,96,98,100,104,107,110^, and 473 patients from 14 studies were included in the AC and CC fixation category^7,18,24,66,71,74,78,86,88,91,92,95,96^. Twelve studies, comprising 380 patients, were included in the Bosworth Screw category^40,42,76,80,89,93,99,102,103,105,106,108^. The tendon graft, synthetic graft, and conservative categories consisted of 8^26,27,31,41,53,61,68,73^, 7^34,37,39,53,62,67,79^, and 9^22,54,59,84,86,87,97,109^ studies with 195, 186, and 329 patients, respectively (Table I). Weighted prevalence of OA per category ranged from 6.7% to 29.3%. The CC fixation category reported the lowest OA prevalence with 6.7% after approximately 3.9 ± 0.9 years of follow-up. Conservative treatment reported the highest OA prevalence with 29.3% after approximately 6.0 ± 2.2 years of follow-up (Table II).

**TABLE II:** Pooled Treatment Categories Characteristics*†

Treatment Category	Pooled Sample Size	Age at Dislocation, yr Mean ± SD	FU, yr Mean ± SD	Pooled Weighted % OA
AC fixation	1,199	37.0 ± 10.5	5.4 ± 1.2	18.8
CC fixation	1,050	36.0 ± 10.4	3.9 ± 0.9	6.7
AC and CC fixation	473	36.0 ± 10.4	3.9 ± 1.6	13.0
Bosworth screw	380	35.8 ± 9.8	5.4 ± 2.7	16.0
Synthetic graft	186	37.0 ± 11.8	6.6 ± 2.0	28.3
Tendon graft	195	40.0 ± 11.1	3.6 ± 1.8	10.3
Conservative	329	36.3 ± 9.0	6.0 ± 2.2	29.3

* Characteristics of pooled treatment categories were presented. Means were presented as numbers with SD and pooled values were presented as numbers or percentages.

† AC = acromioclavicular, CC = coracoclavicular, FU = follow-up, and OA = osteoarthritis.

**Fig. 2:**
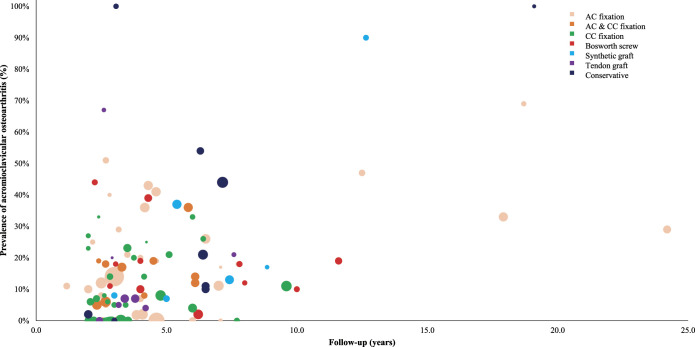
Scatterplot describing the acromioclavicular osteoarthritis prevalence by follow-up duration according to different treatment categories.

**Fig. 3:**
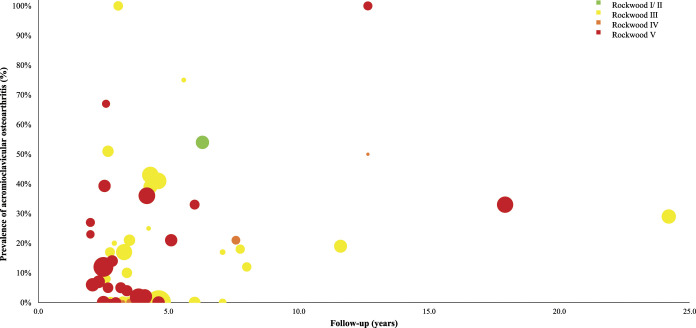
Scatterplot describing the acromioclavicular osteoarthritis prevalence by follow-up duration according to different Rockwood types.

A clear, nonstatistical association was observed between AC OA prevalence and follow-up duration. Studies with a longer follow-up had a higher prevalence of OA. No clear differences were observed between treatment categories or Rockwood types (Fig. 2 and Fig. 3).

### AC OA Prevalence in the Injured and Contralateral Shoulder

**Fig. 4:**
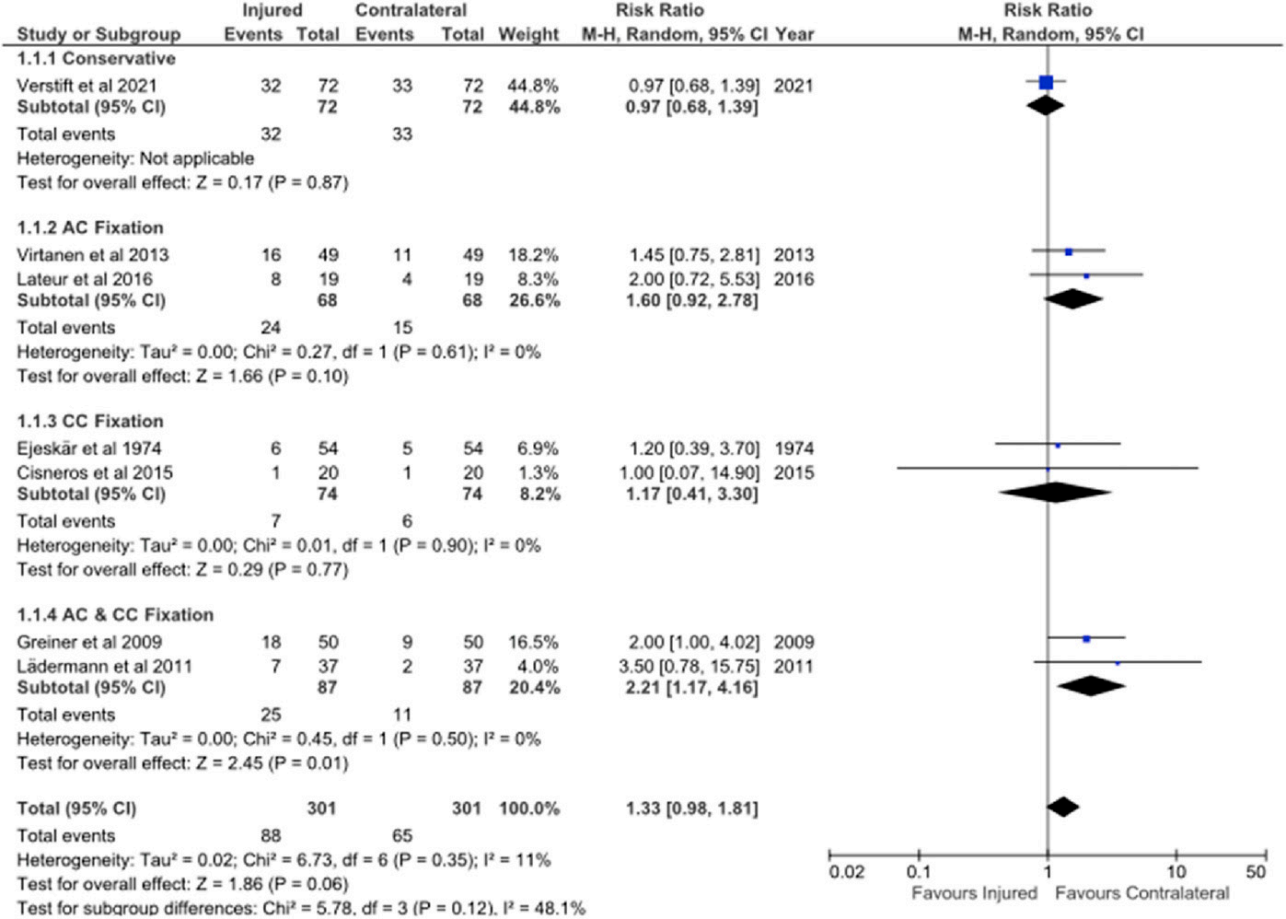
Meta-analysis comparing the acromioclavicular osteoarthritis prevalence of the injured and the uninjured, contralateral shoulder.

Seven studies reported on the AC OA prevalence in the contralateral, uninjured shoulder. A meta-analysis demonstrated no significant difference (p = 0.120) when comparing total prevalence of OA in the injured shoulder to the prevalence of OA in the contralateral shoulder. Subanalysis demonstrated a higher OA prevalence in the injured shoulder after AC and CC fixation (p = 0.010) (Fig. 4).

### Definition of AC OA

Only 23 studies (24.5%) elaborated on the used definition and classification system for AC OA. Most common used definition was diagnosis of AC OA based strictly on radiographic changes in the AC joint compared with preoperatively and compared with the contralateral side (52.2%). Severity was scored as “absent, mild, moderate, and severe.” Other definitions used for diagnosis were the Modified Kellgren-Lawrence classification^111^ (8.7%) and diagnosis of AC OA based on radiographic and clinical results (39.1%) (Table III, Appendix 2).

**TABLE III:** Summary of Mentioned AC OA Definition or Classification System*

	N/N total (%)
Mentioning of AC OA definition or classification	23/94 (24.5)
Types of used classification systems	
Modified Kellgren-Lawrence classification system for AC OA	2/23 (8.7)
Strictly radiographic classification	12/23 (52.2)
Radiographic and clinical classification	9/23 (39.1)

* Summary of the number of included articles mentioning the used definition or classification system for acromioclavicular (AC) osteoarthritis (OA) are presented in frequencies (N) with the frequencies in parentheses. Types of classification systems are presented. Strictly radiographic classification system is based on radiographic changes in the AC joint compared to preoperatively and to the contralateral side. Radiographic and clinical classification system is based on the radiographic and clinical changes in the AC joint compared with preoperatively and compared with the contralateral side.

## Discussion

The major finding was that the he pooled AC OA prevalences of the 7 treatment categories ranged from 6.7% for the CC fixation surgical group to 29.3% for the conservative treatment group. The prevalence of AC OA was primarily influenced by follow-up duration, which differed between treatment groups, instead of treatment type. No differences in the OA prevalence between the surgical and conservative treatment categories were observed. These observations might have been influenced by the heterogeneity of the study characteristics and the various diagnostic modalities used to diagnose AC OA.

Currently, numerous different treatments are used to treat an AC dislocation. If a significant difference in prevalence of postoperative AC OA between treatment options for AC dislocation would have been observed, it could be beneficial to prioritize that treatment, dependent on other factors including treatment durability, cost, and safety. This study demonstrated that the pooled prevalence of AC OA of the different treatment categories ranged from 6.7% to 29.3%, but a clear statistical or nonstatistical difference was not observed. This might have been caused by the high heterogeneity of reported data and used diagnostic modalities for AC OA.

In addition, the prevalence of AC OA seemed to increase as follow-up duration increased. Only the tendon graft treatment category did not contribute to this association, as in the tendon graft category, AC OA prevalence was relatively high when attributing it to its short mean follow-up duration (pooled AC OA prevalence: 10.3%; mean follow-up: 3.6 years).

The comparisons of AC OA prevalence rates between the various treatment categories were affected by the difference in follow-up durations, making hard conclusions on the differences in rates unfeasible. However, the illustrated graphs did not demonstrate a strong difference in postoperative AC OA between the treatment categories. Two radiographic studies by Bonsell et al. and Pennington et al. both reported a 45% AC OA prevalence in the healthy population, which was even higher than the pooled prevalences reported by this study^9,10^. In addition, our meta-analysis, comparing total prevalence of AC OA in the injured shoulder with the prevalence in the contralateral shoulder, found no significant differences. Based on the available data, this study did not conclude that treatment of AC dislocation should be made based on the risk of postoperative AC OA. Moreover, it is important to emphasize that AC OA is not the only radiological change that can occur after AC dislocation. Verstift et al.^22^ found a high prevalence of AC osteolysis and AC deformations after AC dislocation RW type 1 and 2 and a low prevalence of AC OA.

The low number of studies reporting the used diagnostic modalities for AC OA can create confusion, as different definitions can be used to diagnose the same pathology. Three distinct definitions for AC OA were observed. The definitions described by Calvo et al. and Kohn et al. differed in levels of OA severity and solely basing diagnosis on radiographic and/or clinical results^6,112^. Further research on AC OA should provide a clear definition and classification for AC OA because this is essential to compare homogenous data. Diagnosis of AC OA solely based on radiographic results may be used, but the frequency of symptomatic patients should always be reported because this solves the ambiguity of asymptomatic or symptomatic AC OA.

Evaluation of the association between AC OA and follow-up duration after the treatment of AC dislocation was burdened by the patient's age acting as a critical confounder. Multiple studies have demonstrated that radiographic, asymptomatic AC OA is the norm in patients older than 40 years and that AC OA prevalence increases with age^8-10,113^. A systematic review by Rossano et al. reported that 70% of MRI images of asymptomatic shoulders showed signs of asymptomatic AC OA and concluded that asymptomatic AC OA is highly prevalent as humans age^8^. As most included studies in this systematic review did not elaborate on symptomatic or asymptomatic postoperative AC OA, caution should be excised when associating risk of postoperative AC OA with a follow-up, as the risk of AC OA is also associated with age.

This systematic review was limited by several factors. First, because of the heterogeneity in treatment options and Rockwood classifications, a quantitative meta-analysis was not feasible. However, to the best of the author's knowledge, this study comprised the largest patient population analyzing AC OA after AC dislocation. In addition, few studies reported on prevalence of AC OA with a follow-up duration of more than 10 years. Therefore, caution should be excised when interpreting results from this study on long-term follow-up. However, the various treatment modalities had a minimal follow-up of >3.6 years. In addition, follow-up duration differed between the treatment modalities, ranging from 3.6 to 6.6 years, which affected the comparability between treatment modality and AC OA prevalence. Third, most of the included studies had a retrospective design, resulting in a lower MINORS score and a higher risk of bias. However, multiple electronic databases were searched, minimizing risk of selection bias. Fourth, the meta-analysis only included retrospective studies of lesser quality limiting the substantiation of our conclusion. Finally, satisfactory or unsatisfactory treatment outcomes were not accounted for when evaluating prevalence of AC OA although the surgical outcome might correlate with the development of postoperative AC OA. However, accounting for this confounding factor was not feasible as none of the included studies mentioned surgical outcome per patient.

Future research should compare rates of AC OA between patients who underwent surgical treatment for AC dislocation and healthy, nonarthritis patients.

## Conclusion

The pooled AC OA prevalences of the 7 treatment categories ranged from 6.7% for the CC fixation surgical group to 29.3% for the conservative treatment group. No difference in AC OA prevalence was found between the injured and contralateral shoulder. Based on the available evidence, treatment choice for AC dislocation should not be influenced by the potential development of AC AO.

### Sources of Funding

No funding was provided in the investigation of this study.

## Appendix

Supporting material provided by the authors is posted with the online version of this article as a data supplement at jbjs.org (http://links.lww.com/JBJSREV/B122). This content was not copyedited or verified by JBJS.
